# Effects of a Dietary Microalgae (*Arthrospira platensis*) Supplement on Stress, Well-Being, and Performance in Water Polo Players: A Clinical Case Series

**DOI:** 10.3390/nu16152421

**Published:** 2024-07-25

**Authors:** Ignazio La Mantia, Antonino Maniaci, Giuseppe Scibilia, Paolo Scollo

**Affiliations:** 1 Department of Medical, Surgical Sciences and Advanced Technologies G.F. Ingrassia, University of Catania, 95100 Catania, Italy; igolama@gmail.com; 2 Department of Medicine and Surgery, University of Enna “Kore”, 94100 Enna, Italy; paolo.scollo@unikore.it; 3 Gynecology and Obstetrics Department, Giovanni Paolo II Hospital, ASP 7, 97100 Ragusa, Italy; giuseppe.scibilia@asp.rg.it; 4 Gynecological Oncology Unit, Ospedale Cannizzaro, 95125 Catania, Italy

**Keywords:** sports performance, athlete’s subjective performance scale, dietary supplements, water polo, creatine phosphokinase (CPK)

## Abstract

Background: A common tactic used by athletes to improve performance, lessen tiredness, and hasten recovery is dietary supplementation. We aimed to assess the role of a microalgae dietary liquid supplement additivated with Copper 22.5% NRV in water polo players’ performance. Methods: Twenty male water polo players were split into two groups: ten (spirulina group) took a twice-daily nutritional supplement containing 15 mL of spirulina liquid extract (titrated in Phycocyanin 1 mg/mL) and additivated with Copper 22.5% NRV for eight weeks, and ten (the placebo group) did not take the supplement. Subjective evaluations were finished using the Athlete’s Subjective Performance Scale (ASPS). Levels of the biomarker creatine phosphokinase (CPK) were also assessed. Results: The spirulina group’s mean total ASPS score increased significantly from baseline to follow-up and was significantly better than that of the placebo group (*p* < 0.001). Conversely, ASPS ratings in the placebo group slightly decreased. A positive correlation between spirulina supplementation and less severe ASPS was found using correlation matrix analysis. However, there was a slight difference in CPK levels from the baseline to the follow-up in the spirulina group. Conclusions: A dietary supplement comprising spirulina and copper may help water polo players’ subjective performance measurements by lowering muscular tension. Larger, randomized controlled trials are yet required.

## 1. Introduction

Dietary supplementation has become a popular strategy among athletes to enhance physical performance, reduce fatigue, and accelerate recovery [1]. Spirulina (*Arthrospira platensis*), a blue-green microalga, has gained attention due to its high protein content, essential amino acids, vitamins, minerals, and antioxidant properties [2,3]. Recent studies have suggested that spirulina supplementation may help prevent exercise-induced oxidative stress, inflammation, and muscle damage [4,5]. Kalafati et al. [6] demonstrated that spirulina supplementation can increase reduced glutathione (GSH) concentration and attenuate exercise-induced lipid peroxidation in moderately trained individuals. Phycocyanin, a compound found in various plant species, has been reported to have antioxidant and anti-inflammatory effects [7]. Copper, an essential trace element, plays a crucial role in energy metabolism, iron absorption, and the synthesis of hemoglobin and myoglobin [8]. Dietary copper intake has been associated with improved athletic performance and recovery [9]. Because of the unique combination of high-intensity aerobic and anaerobic demands and the additional complexity of the aquatic environment, water polo is particularly selected for nutraceutical supplementation [10,11,12]. Players of water polo encounter unique physiological difficulties, including extended submersion in water, frequent, intense sprints, and a requirement for upper body strength and stamina [13]. Because of these characteristics, water polo players present an intriguing group to research how nutritional supplements affect performance and recuperation. Furthermore, there has been a lot of research on the effects of nutritional supplements on land-based sports but not as much on aquatic sports like water polo [14,15,16,17]. Adequate nutrition is crucial for athletes in high-intensity sports like water polo, as they face unique challenges in meeting their energy and nutrient needs, particularly during periods of intense training and competition [18]. The use of botanical supplements in sports has increased in recent years, driven by athletes’ desire for natural performance enhancers, but concerns about supplement quality and potential contamination underscore the need for rigorous research on their efficacy and safety [19]. This study intends to close a gap in the literature by concentrating on water polo players and providing insights that would be especially helpful to athletes in aquatic sports. Spirulina, a blue-green algae, has gained attention in sports nutrition due to its rich nutrient profile and potential health benefits, including antioxidant, anti-inflammatory, and immune-boosting properties [20]. Spirulina’s potential to enhance athletic performance may be attributed to its antioxidant properties, which could help combat exercise-induced oxidative stress and inflammation [21]. Furthermore, spirulina’s protein-rich content and favorable amino acid profile contribute to it being an attractive supplement for athletes looking for aid in muscle recovery and adaptation [22]. This clinical case series aims to investigate the effects of a dietary supplement containing spirulina liquid extract and copper on subjective measures of asthenia, muscular efficiency, fatigue reduction, and sports performance in water polo players. By exploring the potential benefits of this dietary supplement containing spirulina and copper, we aimed to provide insights into its effectiveness in supporting athletic performance, reducing fatigue, and minimizing muscle damage in water polo players. The findings of this clinical case series may contribute to the development of targeted nutritional strategies for athletes engaging in high-intensity sports.

## 2. Materials and Methods

### 2.1. Study Design and Participants

This clinical case series was conducted as an observational study following the Strengthening the Reporting of Observational Studies in Epidemiology (STROBE) guidelines [23]. A total of 20 male participants were recruited and divided into two groups: 10 water polo players who consumed the dietary supplement containing spirulina and copper (spirulina group) and 10 patients who did not use the supplement (placebo group). The study was approved by the local ethics committee, and all participants provided written informed consent before enrollment (n.19912021/PO). Inclusion criteria for the water polo players were (1) age between 18 and 35 years, (2) a minimum of 3 years of competitive water polo experience, and (3) regular training of at least 5 sessions per week. Exclusion criteria included (1) current use of other dietary supplements, (2) presence of chronic diseases or musculoskeletal injuries, and (3) use of medications that could affect the study outcomes.

### 2.2. Training Regimen and Activity Management

During the eight-week study period, both groups adhered to the same structured training routine. Throughout the trial, all participants were told to continue living their regular lives and refrain from beginning any new physical activity. In order to document any physical activity they engaged in outside of team training, participants filled out weekly activity logs. Training session attendance was tracked. To maintain constant weekly volumes, any sessions missed (due to minor illnesses or injuries) were made up with individual training. During the study period, both groups played in the same number of competitive matches (an average of one match per week).

### 2.3. Dietary Assessment and Control

At the beginning of the study, all participants got basic nutritional advice, which advised them to continue with their regular eating routines for the duration of the study. We used the Food Frequency Questionnaire (FFQ) [24] and 3-Day Food Records [25] to gather information about the participants’ dietary habits, even though stringent dietary control was not applied because of logistical limitations. A validated FFQ was completed by each participant, both at baseline and at the conclusion of the eight-week research period. This gave us the ability to calculate the total amount of nutrients consumed and spot any notable dietary pattern changes that occurred throughout the trial. Furthermore, at baseline, week 4, and week 8, participants were required to complete 3-day dietary records, which comprised two weekdays and one weekend day. Further specific details regarding food selections and serving quantities were supplied by these documents. Nutrition analysis software was used to estimate calorie intake, macronutrient distribution, and critical micronutrient intake using the FFQs and food records.

### 2.4. Supplementation Protocol 

The spirulina group consumed a dietary supplement containing 15 mL of spirulina liquid extract (titrated in Phycocyanin 1 mg/mL) (*Arthrospira platensis*) (BluVIS^®^, Originy S.r.L., Caltagirone, Italy) and additivated with Copper 22.5% NRV twice a day for 8 weeks. The placebo group did not receive any supplement during the study period. The supplement was to be taken twice a day by participants in the spirulina group, with specified scheduling tied to their training sessions. They took the first 15 mL dose 30 min prior to the morning session and the second 15 mL dose 30 min following the conclusion of the evening session on training days. On non-training days, participants were instructed to stick to a similar schedule and take the doses with breakfast and dinner. Each spirulina vial was mixed with 250 mL of water or fruit juice and consumed right away, according to the participants’ instructions. Participants were provided a supplement intake journal to record the time of each dose in order to track compliance. Weekly check-ins were held to gather these logs and deliver the supplements for the following week. Based on the number of empty vials returned and the recorded doses, compliance was determined. The placebo group followed the same protocol as the spirulina liquid but received a placebo vial that was identical in look and flavor to preserve the study’s double-blind design. The vials containing spirulina and placebo were labeled with codes and packaged in a similar manner to prevent participants or researchers from being able to tell them apart during the study. Both groups maintained their regular training routines and dietary habits throughout the study.

### 2.5. Assessment Protocol

At baseline and after 8 weeks of supplementation, participants completed subjective assessments using visual analog scales (VASs) and specific questionnaires for asthenia, muscular efficiency, fatigue reduction, and sports performance. The VAS scores ranged from 0 to 10, with higher scores indicating better outcomes. In addition, we used a modified version of the ASPS questionnaire specific to assessing subjective sport performance [26] (Supplementary File S1). This 15-item survey covers a wide range of performance-related topics and offers insightful information about the athletes’ perceptions of their own talents and experiences. Every question on the ASPS focuses on a different facet of athletic performance; these encompass anything from general performance and sport-specific skills to motivation, recuperation, and mental focus. Each item is rated by participants on a scale of 1 to 10 (overall ASPS score ranges from 15 to 150), with higher scores typically denoting higher satisfaction or better performance. The questionnaire looks at technical issues like maintaining good form throughout tiredness as well as physical traits like strength, power, speed, agility, and endurance. It also explores psychological and emotional aspects, such as motivation, focus, ability to function under pressure, and self-assurance. The ASPS also evaluates how well the athletes believe they are recovering, how well they follow their training plans, and how well they are performing overall in relation to their own objectives. At baseline, the ASPS questionnaire was initially collected a week before the supplementation. On the last day of supplementation or within 24 h of the last dose, the ASPS questionnaire was administered a second time throughout the eight-week follow-up. We performed a first blood test after a 12-h overnight fast, during the same session as the baseline ASPS questionnaire. After an overnight fast of 12 h and 24–48 h after the last supplement administration, blood samples were taken for post-intervention. The blood tests and questionnaires were all administered in the morning between the hours of 7:00 and 10:00 in order to account for fluctuations in physiological markers throughout the day. Creatine phosphokinase (CPK) levels are a biomarker of muscle stress and damage. The samples were analyzed in a certified laboratory using standard protocols. In addition, we evaluated each player’s performance in-depth by the coach (Supplementary File S2). The head coach of the squad, who has vast experience analyzing water polo performance, conducted this evaluation by assessing technical talents; comprehension and application of tactics; swimming performance, such as speed, endurance, and explosive power; overall effectiveness of the team’s play; and performance consistency.

### 2.6. Statistical Analysis

Descriptive statistics were used to summarize the participants’ characteristics and study outcomes. Continuous variables were presented as means ± standard deviations, while categorical variables were reported as frequencies and percentages. The Shapiro–Wilk test was used to assess the normality of the data distribution. Comparisons between the spirulina and placebo groups were performed using independent *t*-tests for normally distributed data and Mann–Whitney U-tests for non-normally distributed data. Within-group changes from baseline to 8 weeks were analyzed using paired *t*-tests or Wilcoxon signed-rank tests, as appropriate. Statistical significance was set at *p* < 0.05. We used a repeated-measures ANOVA to assess the impact of spirulina supplementation on ASPS severity over time. Group (spirulina vs. placebo) constituted the between-subjects factor, while time constituted the within-subjects component in order to investigate the direct impact of group and changes in ASPS severity over time. All analyses were conducted using SPSS software (version 29.0, IBM Corp., Armonk, NY, USA) and Jamovi (Jamovi, Version 2.54, Retrieved from https://www.jamovi.org (accessed on 1 June 2024)).

## 3. Results

Age, body mass (BM), height, fat mass percentage (FM%), fat mass in kilograms (FM kg), and lean body mass (LBM) were among the demographic and anthropometric traits that were comparable between the spirulina and placebo groups, with no statistically significant changes (*p* > 0.05) (Table 1). 

With average attendance rates of 95.3% for the spirulina group and 94.8% for the placebo group (*p* = 0.87), both groups demonstrated great compliance. Analysis of the FFQs and 3-day food records revealed no statistically significant differences in energy intake, macronutrient distribution, or key micronutrient intake between the spirulina and placebo groups at baseline or throughout the study period (*p* > 0.05 for all comparisons). Both groups maintained relatively consistent dietary patterns throughout the 8-week study (Table 2). 

**Figure 1:**
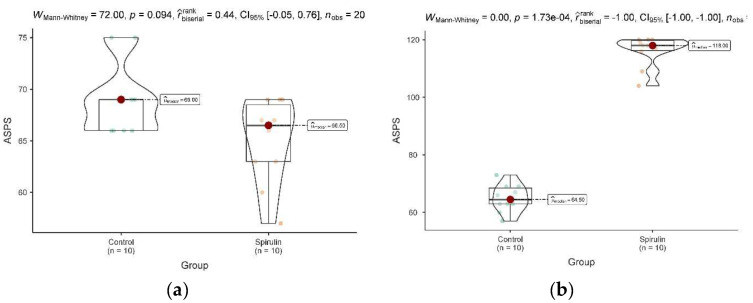
Violin plots comparing ASPS baseline outcomes between spirulina and control (placebo) groups. Changes in ASPS over the study period are shown. These figures illustrate the mean ASPS for both spirulina and placebo groups at baseline (week 0) (**a**) and end of the study (week 8) (**b**). The *y*-axis represents the ASPS score, ranging from 0 to 100, where higher scores indicate better performance. Error bars represent standard deviation. A slight but not significant difference was found at baseline (**a**). The spirulina group shows a steeper increase in ASPS compared to the placebo group, particularly from baseline to week 8 (**b**). (**b**): Athlete’s ASPS trajectory over the 8-week study period. This figure highlights the variability in individual responses while demonstrating the overall trend of greater improvement in the spirulina group.

At baseline, the placebo group slightly outperformed the spirulina group in terms of mean scores for the majority of individual items, as well as the overall ASPS score (69.0 ± 3.46 vs. 64.56 ± 4.14). Nevertheless, there was no statistically significant difference (*p*-value = 0.154) (Figure 1).

**Figure 2:**
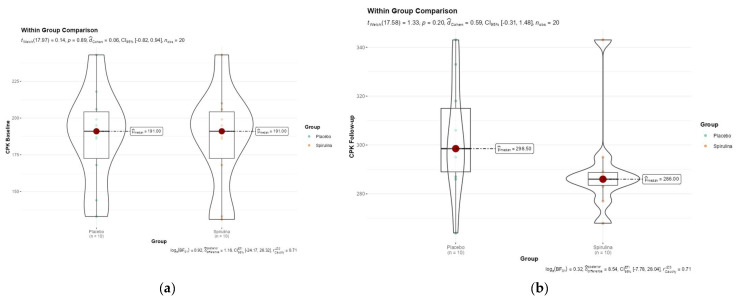
(**a**,**b**) Violin plot for spirulina and placebo group comparison at baseline and 8-week follow-up. No significant differences were found. The placebo group’s mean CPK level rose to 303.1 ± 24.66 U/L at follow-up (*p* < 0.001), suggesting that they underwent more muscular stress during activity (**b**). Similarly, the mean CPK level in the spirulina group rose to 289.3 ± 21.42 U/L U/L (*p* = 0.001). In addition, after intergroup analysis of the two groups, a slight but not significant change in CPK level was recorded (*p* = 0.115). Regardless of supplementation status, both the spirulina and placebo groups had higher CPK levels, indicating persistent muscle injury. These data imply that the water polo players’ rigorous daily training schedule was a significant contributor to muscle stress.

However, the ASPS scores of the spirulina group were considerably higher than those of the placebo group at the follow-up. Individual question mean scores for the spirulina group were 7.7–8.1 (SD: 0.74–1.03), whereas the placebo group’s mean scores were 4.0–4.7 (SD: 0.95–1.35). The group treated with spirulina had a significantly higher overall ASPS score (116.1 ± 5.36) compared to the placebo group (65.0 ± 4.74) (*p* < 0.001). As demonstrated by the increase in mean total ASPS score from 64.56 ± 4.14 at baseline to 116.1 ± 5.36 at follow-up and the consistently higher mean scores for individual questions (Q1-Q15) at follow-up (7.7 to 8.1, SD: 0.74 to 1.03) compared to baseline (3.89 to 4.78, SD: 0.95 to 1.37), the spirulina group demonstrated a significant improvement in athletic performance from baseline to follow-up (*p* < 0.001). Conversely, the placebo group showed a slight decline in athletic performance (4.3 to 5.0, SD: 0.79 to 1.17) to follow-up (4.0 to 4.7, SD: 0.95 to 1.35) (*p* = 0.069). The repeated-measures ANOVA revealed a significant main influence of time (F(2,36) = 15.32, *p* < 0.001), indicating that there was a significant shift in ASPS scores for both groups over the course of the study. More importantly, we discovered that group and time had a significant interaction (F(2,36) = 8.76, *p* = 0.001), suggesting that there were variations in the changes in ASPS severity over time between the spirulina and placebo groups. Throughout the course of the trial, the coach assessment (CQ) found significant disparities between the spirulina and placebo groups. The spirulina group exhibited a more significant improvement at follow-up (148.2 ± 12.7) compared to a small gain in the placebo group (137.9 ± 13.5) (*p* < 0.001), despite the fact that both groups had similar baseline scores (spirulina: 132.5 ± 15.3; placebo: 134.1 ± 14.8; *p* = 0.233). Interesting results were obtained when the placebo and spirulina groups’ baseline and follow-up CPK levels were compared. Baseline CPK levels were similar in both groups: 186.8 ± 34.29 U/L for the spirulina group and 187.3 ± 32.56 U/L for the placebo group (*p* = 0.795) (Figure 2a). 

### Predictors of Muscular Stress and Spirulina Consumption

**Figure 3:**
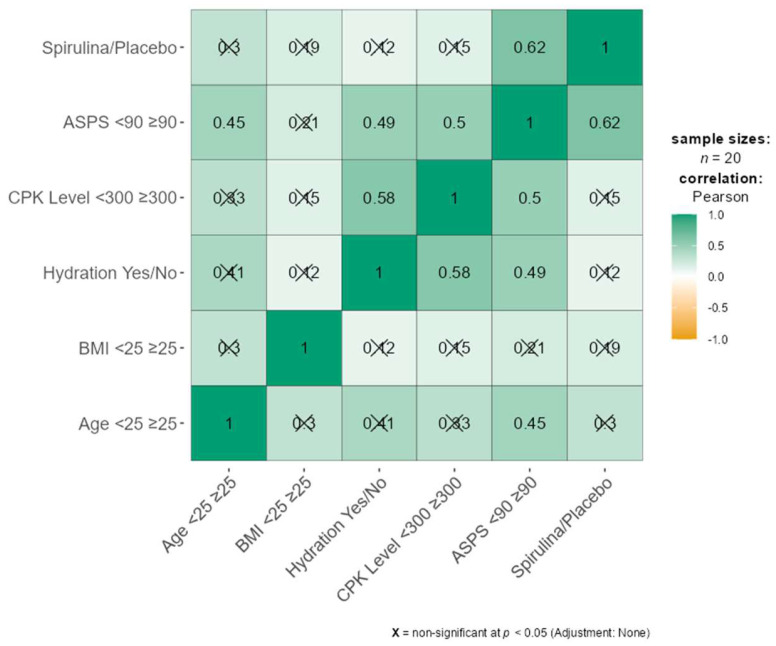
Correlation matrix of dependent variables and spirulina consumption.

The moderately positive connection (r = 0.62) between ASPS severity and spirulina consumption is one of the most remarkable findings (Figure 3). Based on ASPS symptoms, it appears that patients who took spirulina were more likely to have reduced muscle stress. There was also a significant connection (r = 0.50) between ASPS severity and CPK levels, suggesting a relationship between laboratory data and subjectively reported stress. Rather, the degree of ASPS was shown to have a moderately favorable connection (r = 0.49) with hydration state and age (r = 0.45). Weaker relationships were observed between age and BMI than with other variables.

## 4. Discussion

The current clinical case series is the first to examine how water polo players’ subjective assessments of their athletic performance and muscle injury were affected by a nutritional supplement that included a liquid extract of spirulina, as well as copper. Our findings confirm that supplementation might be advantageous for lowering muscular strain and enhancing subjective performance evaluations [27,28]. A noteworthy discovery was the enhancement in the ASPS ratings between the spirulina and placebo groups. From baseline to follow-up, the spirulina group experienced an amelioration in total ASPS score, suggesting improved perceived athletic performance. The ASPS scores of the placebo group, on the other hand, slightly decreased. These outcomes are in line with other research that found that spirulina supplementation had antioxidant and ergogenic benefits for athletes [29]. A somewhat favorable link between the consumption of spirulina and decreased ASPS severity was also found by the correlation matrix analysis, indicating that spirulina supplementation may help reduce muscular tension and enhance subjective performance. Furthermore, a slight but not significant relationship was discovered between the severity of ASPS and the levels of the muscle injury biomarker CPK. This result validates the use of ASPS as a subjective metric for measuring muscle stress that is consistent with objective laboratory data. Interestingly, from baseline to follow-up, both study groups significantly increased CPK levels after muscle stress. It is significant to remember that for the course of the trial, both the spirulina and placebo groups had higher CPK levels, indicating persistent muscle injury. Regardless of supplementation status, these data imply that the water polo players’ rigorous daily training schedule was a significant contributor to muscle stress. Our findings are similar to earlier studies on the effects of high-intensity sports on muscle injury, which have repeatedly demonstrated that dietary interventions, while maybe helpful, fall short of completely mitigating the damage that exercise causes to muscles [30,31].

Our study’s reliance on a single biomarker (CPK) may have limited our ability to detect the full range of physiological effects of spirulina supplementation; future studies should consider incorporating a more comprehensive panel of biomarkers to provide a more holistic view of the supplement’s impact on athlete physiology. Yet, the spirulina group showed a slight mean value compared with the placebo group at intergroup analysis. These findings imply that long-term spirulina supplementation may offer protection against the deterioration of muscles caused by exercise, presumably because of its anti-inflammatory and antioxidant characteristics [6,7,8]. The association matrix also demonstrated the significance of age and hydration state as somewhat positive indicators of the severity of ASPS. Age may have an impact on how the body reacts to stress brought on by exercise, and staying properly hydrated is essential for both peak athletic performance and recuperation. These findings highlight the necessity of a thorough strategy for athlete nutrition and recuperation that takes into account variables other than dietary supplements [32,33]. It is critical to recognize the study’s limitations, which include the observational approach and small sample size. The absence of blinding and randomization could lead to biases. Furthermore, the study’s focus on male water polo players limited the findings’ applicability to female athletes and other sports. Larger, randomized controlled trials should be used in future studies to examine the effects of supplementing with spirulina and copper on muscle injury and athletic performance in a variety of sports and demographics. A number of limitations should be taken into account, even though this clinical case series offered some initial evidence in favor of the possible advantages of a dietary supplement containing spirulina and copper in lowering muscle stress and improving subjective performance measures in water polo players. It is crucial to remember that although we took steps to learn about the participants’ food intake, we did not impose stringent dietary controls. This restriction should be taken into account when interpreting our findings, and in order to further isolate the effects of the supplement, future research should strive for stricter dietary controls. The results emphasize the value of specific dietary approaches to enhance athletic performance and recuperation. Although the subjects of this study were water polo players, athletes participating in other sports, especially those requiring high-intensity interval training, sustained endurance, or substantial upper body exertion, may also benefit from spirulina and copper supplementation. It is crucial to remember that the particular results seen in water polo may be influenced by the special combination of the aquatic environment and physical demands of the sport. Similar physiological needs may be seen in sports like swimming or other aquatic activities. Although the effects may vary in size, land-based sports with high-intensity interval components (such as basketball and soccer) may also be beneficial. To determine whether there are any response variations specific to a particular sport and to validate the effectiveness of this supplement combination in a variety of sports, more research is required. We acknowledge that body weight may have an impact on the effectiveness of spirulina supplements, even though our trial only employed a fixed dose of the supplement. If body weight has an impact on supplement efficacy, future studies should investigate weight-based dosage or stratifying analysis based on participant body weight. This observation points to a possible direction for more individualized spirulina supplementation strategies for sportsmen. Although the main focus of our study was on the effects of spirulina, more research should be done to see whether spirulina and copper in the supplement can work in concert. More focused research is needed on the function of copper in energy metabolism and its potential to improve sports performance when paired with other nutrients [34,35]. Our approach, which focused on the effects of spirulina, did not provide a thorough analysis of the separate and combined effects of spirulina and copper. In order to isolate the effects of each component and any potential synergistic interactions between them, future research should think about utilizing a factorial design [36]. Our study design’s limitations in exploring these possible synergistic effects draw attention to the necessity of conducting more intricate, multi-arm trials in the field of sports nutrition. These investigations may yield important information on the potential interactions between various micronutrients and bioactive substances in supplements and their effects on recovery and athletic performance [37]. While our findings are consistent with those of Kalpana et al. [38] and point to possible advantages of the spirulina-based supplement, we are unable to conclusively link spirulina to these effects or rule out the possibility of copper playing a role. This emphasizes the significance of taking into account nutritional supplements’ entire composition in studies on sports performance, as stressed by Maughan et al. [1]. This study has a limitation in that, even with the inclusion of VAS and ASPS questionnaires and a coach assessment, our evaluation methods might not fully capture the complexities of performance in water polo. Future research would benefit from adding physical performance tests specific to the sport, like swimming speed, ball-throwing velocity, and assessments of muscle strength and endurance, to complement these measures and provide a more thorough and objective evaluation of athletes’ performance in the context of water polo. It is important to note that our study design, which used a fixed dose of spirulina for all participants in the supplementation group, did not allow for the examination of a dose–response relationship. Future research could explore this aspect by incorporating varying doses of spirulina supplementation. In addition, to determine broader applications, we advise future research to investigate these effects in a variety of sports populations. 

## 5. Conclusions

A dietary supplement containing spirulina (titrated in Phycocyanin 1 mg/mL) and copper may have beneficial effects on reducing muscle stress and improving subjective performance measures in water polo players. More investigation is required to validate these findings and clarify the underlying mechanisms of action. Before introducing dietary supplements into their training regimens, athletes and sports professionals should carefully assess their particular demands and speak with expert healthcare providers.

## Figures and Tables

**Table 1:** Values are presented as mean ± standard deviation. BM, body mass; FM, fat mass; LBM, lean body mass; ASPS, Athletic Self-Performance Survey; CQ, coach questionnaire.

Characteristic	Placebo (n = 10)	Spirulina (n = 10)	*p*-Value
Age (year)	24.7 ± 3.1	25.4 ± 2.9	0.295
Weight (kg)	75.5 ± 7.7	78.2 ± 14.1	0.289
Height (cm)	181.9 ± 5.3	182.1 ± 4.9	0.721
FM (%)	18.7 ± 4.6	19.5 ± 5.1	0.567
FM (kg)	18.1 ± 5.9	19.5 ± 7.2	0.532
LBM (kg)	77.4 ± 8.1	78.7 ± 8.5	0.578
Baseline CPK (U/L)	187.3 ± 32.56	186.8 ± 34.28	0.620
Total ASPS Score (Baseline)	69.0 ± 3.5	66.9 ± 4.1	0.154
CQ	134.1 ± 14.8	132.5 ± 15.3	0.233
Years of experience	10.2 ± 2.8	10.5 ± 3.1	0.938
Training volume (hours/week)	18.5 ± 2.3	19.1 ± 2.5	0.142
Competitive level			
National	6 (60%)	7 (70%)	0.138
Regional	4 (40%)	3 (30%)	

**Table 2:** The data are shown as mean ± standard deviation. *p*-values show group comparisons at week 8. From baseline to week 8, there were no significant within-group changes for any dietary component (*p* > 0.05 for all comparisons).

Dietary Component	Spirulina Group		Placebo Group		*p*-Value
	Baseline	Week 8	Baseline	Week 8	
Energy (kcal/day)	2785 ± 312	2798 ± 305	2803 ± 298	2815 ± 287	0.871
Macronutrients (% of total energy)					
Carbohydrates	52.3 ± 4.1	52.1 ± 3.8	51.8 ± 3.9	52.0 ± 4.2	0.924
Proteins	21.7 ± 2.3	21.9 ± 2.4	22.1 ± 2.5	21.8 ± 2.6	0.883
Fats	26.0 ± 3.2	26.0 ± 3.1	26.1 ± 3.0	26.2 ± 3.3	0.952

## Data Availability

The original contributions presented in the study are included in the article/Supplementary Materials, further inquiries can be directed to the corresponding author.

## Supplementary Materials

The following supporting information can be downloaded at: https://www.mdpi.com/article/10.3390/nu16152421/s1, Supplementary File S1: ASPS questionnaire. Legend: 15–30: Poor overall performance; 31–60: Below average overall performance; 61–90: Average overall performance; 91–120: Above average overall performance; 121–150: Excellent overall performance.; Supplementary File S2: Coach assessment. Legend Rating Scale: 1-2: Poor; 3–4: Below Average 5–6: Average; 7–8: Good; 9–10: Excellent; Total Score: ____ / 180.
