# Supplementary material for: Effects of a Dietary Microalgae (Arthrospira platensis) Supplement on Stress, Well-Being, and Performance in Water Polo Players: A Clinical Case Series
Source: Nutrients. 2024 Jul 25;16(15):2421. doi: 10.3390/nu16152421 (PMC11314195; doi:10.3390/nu16152421)
Supplement: Supplementary file 1 [file nutrients-16-02421-s001.zip › nutrients-3094817-supplementary.pdf]

Question	Rating Scale	ASPS Score
1. On a scale of 1 to 10, how would you rate your overall athletic performance in the past week?	1 = poor, 10 = excellent	
2. How would you rate your endurance during training sessions and competitions?	1 = poor, 10 = excellent	
3. How would you rate your speed and agility in your specific sport?	1 = poor, 10 = excellent	
4. How would you rate your strength and power in relation to your sport's requirements?	1 = poor, 10 = excellent	
5. How would you rate your mental focus and concentration during training and competitions?	1 = poor, 10 = excellent	
6. How quickly do you recover between intense training sessions or competitions?	1 = very slow, 10 = very fast	
7. How would you rate your overall energy levels during training and competitions?	1 = very low, 10 = very high	
8. How would you rate your ability to perform under pressure during crucial moments in competitions?	1 = poor, 10 = excellent	
9. How satisfied are you with your current level of technical skills in your sport?	1 = not satisfied, 10 = extremely satisfied	
10. How would you rate your ability to maintain proper form and technique during fatigue?	1 = poor, 10 = excellent	

Question	Rating Scale	ASPS Score
11. How would you rate your flexibility and range of motion in relation to your sport's requirements?	1 = poor, 10 = excellent	
12. How well do you adhere to your training and recovery routines?	1 = poor adherence, 10 = excellent adherence	
13. How would you rate your overall confidence in your athletic abilities?	1 = low confidence, 10 = high confidence	
14. How motivated are you to continuously improve your sports performance?	1 = not motivated, 10 = highly motivated	
15. How would you rate your overall sports performance compared to your personal goals and expectations?	1 = well below expectations, 10 = well above expectations	
		Total ASPS Score:

Supplementary file S1. MODIFIED ASPS. **Legend:** 15-30: Poor overall performance; 31-60: Below average overall performance; 61-90: Average overall performance; 91-120: Above average overall performance; 121-150: Excellent overall performance.

Supplementary file S2. Coach assessment. Legend Rating Scale: 1-2: Poor; 3-4: Below Average 5-6: Average; 7-8: Good; 9-10: Excellent; Total Score: ____ / 180

Category	Performance Indicator	Rating (1-10)
**Technical Skills**	Passing accuracy	
	Shooting precision	
	Ball handling	
	Defensive positioning	
**Tactical Understanding**	Reading the game	
	Decision making	
	Positioning	

Category	Performance Indicator	Rating (1-10)
	Team play contribution	
**Physical Performance**	Swimming speed	
	Endurance	
	Explosive power	
	Agility in water	
**Mental Aspects**	Focus during matches	
	Resilience under pressure	
	Communication with teammates	
**Overall Performance**	Consistency throughout study period	
	Impact on team's performance	
	Improvement from baseline	
